# Suppression of Stim1 reduced intracellular calcium concentration and attenuated hypoxia/reoxygenation induced apoptosis in H9C2 cells

**DOI:** 10.1042/BSR20171249

**Published:** 2017-11-23

**Authors:** Fei He, Qianfu Wu, Banglong Xu, Xiaocheng Wang, Jixiong Wu, Li Huang, Jing Cheng

**Affiliations:** 1 Department of Cardiology, Second Affiliated Hospital of Anhui Medical University, No. 678 of Furong Road, Hefei, Anhui, China; 2 Department of Geriatrics Medicine, Shanghai Municipal Hospital of Traditional Chinese Medicine, No. 274 of Middle Zhijiang Road, Jingan District, Shanghai, China; 3 School of Nursing, Anhui University of Chinese Medicine, No. 103 of Middle Meishan Road, Hefei, Anhui, China

**Keywords:** Apoptosis, Calcium overload, Myocardial reperfusion injury, Orai1, STIM1

## Abstract

Objective: Previous studies have demonstrated Stromal interaction molecule 1 (STIM1)-mediated store-operated Ca^2+^ entry (SOCE) contributes to intracellular Ca^2+^ accumulation. The present study aimed to investigate the expression of STIM1 and its downstream molecules Orai1/TRPC1 in the context of myocardial ischemia/reperfusion injury (MIRI) and the effect of STIM1 inhibition on Ca^2+^ accumulation and apoptosis in H9c2 cardiomyocytes subjected to hypoxia/reoxygenation (H/R).

Methods: Expression of STIM1/Orai1/TRPC1 was determined by RT-PCR and Western blot in mice subjected to MIRI and H9C2 cardiomyocytes subjected to H/R. To knock-down STIM1, H9C2 cardiomyocytes was transfected with Stealth SiRNA. Apoptosis was analyzed by both flow cytometry and TUNEL assay. Cell viability was measured by MTT assay. Intracellular Ca^2+^ concentration was detected by laser scanning confocal microscopy using Fluo-3/AM probe. Furthermore, the opening of mitochondrial permeability transition pore (mPTP) was assessed by coloading with calcein AM and CoCl_2_, while ROS generation was evaluated using the dye DCFH-DA in H9C2 cardiomyocytes.

Results: Expression of STIM1/Orai1/TRPC1 significantly increased in transcript and translation level after MIRI *in vivo* and H/R *in vitro*. In H9C2 cardiomyocytes subjected to H/R, intracellular Ca^2+^ accumulation significantly increased compared with control group, along with enhanced mPTP opening and elevated ROS generation. However, suppression of STIM1 by SiRNA significantly decreased apoptosis and intracellular Ca^2+^ accumulation induced by H/R in H9C2 cardiomyocytes, accompanied by attenuated mPTP opening and decreased ROS generation. In addition, suppression of STIM1 increased the Bcl-2/Bax ratio, decreased Orai1/TRPC1, and cleaved caspase-3 expression.

Conclusion: Suppression of STIM1 reduced intracellular calcium level and attenuated hypoxia/reoxygenation induced apoptosis in H9C2 cardiomyocytes. Our findings provide a new perspective in understanding STIM1-mediated calcium overload in the setting of MIRI.

## Introduction

Reperfusion therapy with either intravenous thrombolysis or coronary revascularization is effective strategy to reduce infarct size and improve clinical outcome after the onset of acute myocardial infarction [1]. However, the restoration of blood to the ischemic myocardium may also cause ischemia/reperfusion (I/R) injury, leading to reduced benefit from reperfusion [2]. Currently, the underlying pathophysiological mechanisms of myocardial I/R injury have not yet been fully elucidated. As an important second messenger, intracellular calcium (Ca^2+^) has demonstrated to be implicated in the signaling networks that regulate pathological conditions, in addition to the process of myocardial contraction. Actually, abundant evidence suggested that intracellular Ca^2+^ overload might participate in the pathogenesis and progress of the I/R injury [3–5].

Recent studies revealed that store-operated Ca^2+^ entry (SOCE) was one of the major mechanisms account for Ca^2+^ entry in virtually all nonexcitable cells and certain excitable cells, containing the cardiomyocytes [6,7]. As the response to initial Ca^2+^ release from endoplasmic reticulum (ER), SOCE promotes the influx of Ca^2+^ from the extracellular space, followed by a sustained raise in cytosolic Ca^2+^ concentration and subsequent activation of numerous signal transduction pathways [8,9]. Emergent evidence suggest that both Stromal interaction molecule 1 (STIM1) and Orai1 are key mediators of SOCE [7,10,11]. It has been demonstrated that SOCE implicated in various pathological conditions of the cardiovascular system, including myocardial I/R injury [11–13]. To the best of our knowledge, there is no direct evidence on the role of STIM1 and Orail1 in the context of myocardial I/R injury. The present study was designed to investigate whether STIM1 exerts protective effect on cardiomyocytes during myocardial I/R injury by modulating the intracellular Ca^2+^ concentration and reducing subsequent cardiomyocytes apoptosis.

## Materials and methods

### Animals

Adult male wild-type C57 BL/6 mice (10–12 weeks old; 26–30 g) were provided by the Anhui Medical University Laboratory Animal Center (SCXK 2006-0015). All animals used in the present study received ethical and humane care. Experimental procedures were conducted in compliance with the National Institutes of Health Guidelines for Care and Use of Laboratory Animals and were approved by the Bioethics Committee of Anhui Medical University.

### Establishment of myocardial I/R injury model in mice

Mice myocardial I/R injury model was produced as described previously [14,15]. In brief, mice were anesthetized with an intraperitoneal injection of a mixture of xylazine (5 mg/kg) and ketamine (100 mg/kg). To establish the *in vitro* myocardial I/R, the left ascending artery (LAD) was occluded using an 8-0 silk suture tied transiently over PE-10 tubing for 30 min, then the knot on the PE-10 tubing was cut. Successful ischemia was determined by the elevation of ST segment and reperfusion was determined by recovery of the elevated ST segment on surface ECG. Sham-operated mice underwent the same surgical procedures with the exception of left anterior descending coronary artery occlusion.

### Measurement of myocardial infarct size

2,3,5-triphenyltetrazolium chloride (TTC) and Evans Blue were obtained from Sigma-Aldrich (St. Louis, MO, U.S.A.). Following 24h reperfusion, myocardial infarct size was evaluated using TTC/Evans Blue stain, as previously described [16,17]. Briefly, the LAD was retied followed by intravenous administration of 2 ml of 2% Evans Blue dye through the jugular vein. Fifteen minutes later, mice were killed and hearts were excised. The left ventricle was frozen at −20°C for 10 min and then sectioned transversely into 2-mm slices and incubated in 1% TTC dissolved in PBS at 37°C for 10 min. Subsequently, each section was immersed in 4% formaldehyde for 24 h before being photographed. Areas of infarct size (INF) and area-at-risk (AAR) were measured digitally using ImageJ software. INF and AAR were expressed as percentages of the left ventricular area (INF/LV and AAR/LV respectively).

### Assessment of apoptosis in the myocardium from mice underwent I/R injury

Cardiomyocyte apoptosis was evaluated using the terminal deoxynucleotidyl transferase-mediated dUTP nick-end labeling (TUNEL) technique as previously described [17]. TUNEL Apoptosis Assay Kit was the product of Roche diagnostics (Mannheim, Germany) and purchased from Sigma-Aldrich (Cat No.11684795910, St. Louis, MO, U.S.A.). Briefly, 50 μl of TUNEL reaction mixture was added on samples, and the slides were incubated in humidified atmosphere for 60 min at 37°C in the dark and then rinsed with PBS (pH 7.4) three times with 5 min. DAPI staining was performed for total nuclei quantification. The percentage of TUNEL (green fluorescence) positive cells in total cells per field was used to quantify cardiomyocytes apoptosis in ischemic myocardium. The myocardial apoptotic index was calculated as the percentage of TUNEL-positive nuclei per 1000 total nuclei, in the infracted border region in ten random high power field (HPF) per mouse (*n* = 6 per group).

### Exposure of H9C2 cells to hypoxia/reoxygenation (H/R)

H9C2 cells were purchased from ATCC (Manassas, VA, U.S.A.), seeded at constant density (10,000/cm^2^) and grown to 80% in DMEM containing 10% fetal bovine serum and antibiotics. Cells were washed with Hank’s balanced salt solution (HBSS) and rendered quiescent in serum-free DMEM for 24 h prior to experiments. To mimic the myocardial I/R *in vitro*, H9C2 cells at 80% confluence were incubated with a glucose-free medium (previously bubbled with gas mixture containing 95% N_2_ and 5% CO_2_) for 6 h at 37°C [18]. Then the cells were provided with fresh medium and then moved to 95% O_2_/5% CO_2_ for reoxygenation. The control plates were kept in the incubator with 95% O_2_/5% CO_2_ at 37°C. Cells were harvested 16 h post-reoxygenation for analysis.

### Synthesis and selection of SiRNA for STIM1

Desalted Stealth RNAi duplexes were designed using Block-iT RNAi Designer (Thermo Fisher scientific, Shanghai, China) against STIM1 (GenBank Accession No. NM_001108496.2). Three different nonoverlapping RNAi duplexes with the following sequences were obtained (Invitrogen):

1. (STIM1_547): 5′- CAGUGAAGAUGAGAAGCUCAGCUUU-3′ (sense),
  5′- AAAGCUGAGCUUCUCAUCUUCACUG-3′ (antisense);
2. (STIM1_664): 5′-CAATTACCATGACCCTACAGTGAAA-3′ (sense),
  5′-UUUCACUGUAGGGUCAUGGUAAUUG-3′ (antisense);
3. (STIM1_696): 5′-CCUUCCAUGGUGAGGACAAGCUUAU-3′(sense),
  5′-AUAAGCUUGUCCUCACCAUGGAAGG-3′ (antisense).

H293 cells were chosen to test transfection and inhibition efficiency of the three sequences. STIM1_547 showed the highest inhibition efficiency at 81.03%, and a 50 nM dose was selected. H9C2 cardiomyocytes were transfected with siRNA duplexes using Lipofectamine RNAi MAX reagent (Invitrogen) according to the manufacturer’s instructions. The cells were used for experiments 48 h after siRNA transfection.

### Assessment of apoptotic cell death in cultured H9C2 Cells

Apoptotic H9C2 cardiomyocytes were measured using both flow cytometry and TUNEL staining. For flow cytometry, cells were harvested and stained with Annexin V-FITC and propidium iodide (PI) (Thermo Fisher Scientific, Shanghai, China) for 20 min at room temperature. The cells were then washed twice with PBS, and the fluorescence was analyzed with CellQuest (BD Biosciences, Franklin Lakes, NJ) software on data obtained from the cell population. For TUNEL assay, H9C2 cardiomyocytes were fixed with 4% neutral buffered formalin solution for 30 min. Then labeling protocol was done according to the manufacturer’s instruction. Apoptotic cells were observed using a fluorescence microscope, and were counted with at least 100 cells from four randomly selected fields in each group.

### Assessment of cell viability in cultured H9C2 cells

Cell viability was assessed using the methylthiotetrazole (MTT) assay kit (Beyotime, China) according to the manufacturer’s instructions [19]. Briefly, cells were cultured in a 96-well plate at a density of 1 × 10^4^ cells/well and incubated for 24 h. The cells were administered with fresh medium and MTT solution (20 μl out of 5 mg/ml) for 4 h followed by incubation with formazan solution (10 μl) for 4 h at 37°C. The optical density (OD) values at 570 nm were measured using Synergy2 microplate reader (Biotek Instruments Inc., Vermont, U.S.A.). Each experiment was repeated six times and the data were expressed as a percentage of the control.

### Real-time quantitative PCR

Total RNA was isolated from myocardial homogenate and cultured H9C2 cardiomyocytes using the TRIzol reagent (Thermo Fisher scientific, Shanghai, China) according to the manufacture’s instructions and stored at −80°C. Quantitative specific RNA expression was performed in one step with SYBR Green Master kit (TOYOBO). All reactions were performed in triplicate in ABI PRISM^®^ 7500 Sequence Detection System (Thermo Fisher scientific, Shanghai, China). After initial denaturation step at 95°C for 5 min, temperature cycling was initiated for 40 cycles of amplification (denaturation at 94°C for 30 s, annealing at 62°C for 30 s, and elongation at 72°C for 32s). The value obtained for the target gene expression were normalized to 18s rRNA and analyzed by the relative gene expression 2^−ΔΔCT^ method. The following primers were used to assess gene expression of STIM1 and 18s rRNA:

STIM1: Forward 5′-TGC CAA GGC TAG CTG TAA CAA-3′,
  Reverse 5′-TCA GGT GAT TGT GGC GAGTC-3′;
Orai1: Forward 5′-AGG TGA TGA GCC TCA ACGAG-3′,
  Reverse 5′-TAA CCC TGG CGG GTA GTCAT-3′;
TRPC1: Forward 5′-AGA TGT GCT TGG GAG AAA TGCT-3′,
  Reverse 5′-CGT TCC ATA AGT TTC TGA CAA CCG-3′;
18s rRNA: Forward 5′-CCT GGA TAC CGC AGC TAGGA-3′,
  Reverse 5′-GCG GCG CAA TAC GAA TGC CCC-3′.

### Western blot analyses

The operation procedure was described in detail elsewhere [17]. The primary antibodies against STIM1, cleaved caspase-3, and β-actin were purchased from Cell Signaling Technology and the antibodies against Orai1, Bcl-2, Bax, and Transient receptor potential channel 1 (TRPC1) from Abcam. The positive protein bands were developed using a chemiluminescent system, and the bands were scanned and quantified by densitometry analysis using an image analyzer Quantity One System (Bio-Rad, Richmond, U.S.A.).

### Intracellular Ca^2+^ concentration measurements by Laser scanning confocal microscopy

To detect intracellular calcium concentration, H9C2 cardiomyocytes were incubated with Fluo-3/AM (Alexis, Invitrogen, U.S.A.) at 37°C for 60 min according to the manufacturer’s protocol which has been described before [18]. Fluorescence was monitored at 528 nm (excitation: 490–500 nm) using a LSM510 confocal laser scanning microscope (Carl-Zeiss, Jena, Germany). Images were acquired and quantitative analysis of laser scanning confocal microscopy was conducted using Image-Pro Plus 6.0 software (Media Cybernetics Inc., Silver Spring, MD, U.S.A.). Values were expressed as IOD and represented as mean ± SEM.

### Mitochondrial permeability assay

To analyze the opening of mitochondrial permeability transition pore (mPTP), H9C2 cardiomyocytes were loaded with 1.0 mM of calcein–acetomethoxy ester (Molecular Probes) and 1.0 mM CoCl_2_ in HBSS (Gibco) for 20 min at 37°C, then the cells were observed and photographed by fluorescence microscopy.

### Measurement of intracellular ROS accumulation

2′,7′-dichlorodihydrofluorescein diacetate (DCFH-DA) was used to detect intracellular ROS level in H9c2 cardiomyocytes. Briefly, H9C2 cardiomyocytes (10^6^ cells/ml) were incubated with 25 μM DCFH-DA in DMEM without FBS at 37°C for 30 min, then washed thrice with PBS. Cells were collected and the 2′,7′-dichlorofluorescein (DCF) fluorescence was observed with microplate reader (Bio-Rad, U.S.A.) at an excitation wavelength of 488 nm and an emission wavelength of 525 nm.

### Statistical analyses

All quantitative data were expressed as mean ± SEM. The differences between groups were analyzed with either independent sample *t*-test or one-way ANOVA followed by Student–Newman–Keuls *post hoc* analyses for pair-wise comparison. *P*<0.05 was considered to be statistically significant.

## Results

### Expression of STIM1, Orai1, and TRPC1 in myocardium after I/R injury

**Figure 1:**
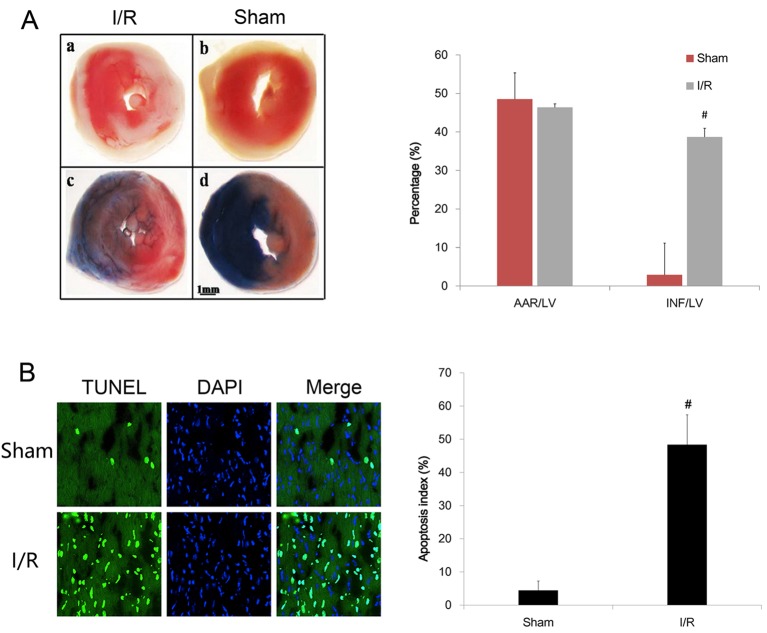
Determination of infarct size and apoptosis index in mice subjected to Sham operation and myocardial I/R injury (**A**) Representative photomicrographs of TTC/Evan Blue dye in heart tissue from mice underwent Sham operation and I/R injury. Infarct size significantly increased following myocardial I/R in mice (6.98 ± 1.59% vs 32.53 ± 3.07%, *t* = − 23.376, *P*<0.001); **#***P*<0.05 vs Sham operation; (**B**) Representative micrographs of left mid-ventricular sections with TUNEL-staining. Apoptosis cells were identified by TUNEL staining(green), and total nuclei by DAPI staining (blue). Apoptosis index significantly increased following myocardial I/R in mice (7.13 ± 2.84% vs 42.53 ± 4.53%, *t* = −20.943, *P*<0.001, *n* = 10 in each group). The results shown are mean ± S.E.M. from three independent experiments; **#***P*<0.05 vs Sham operation.

To verify the *in vivo* model of myocardial I/R injury in mice, infarct size was evaluated by TTC/Evans Blue staining and myocardial apoptosis was assessed by TUNEL assay 24 h after the end of reperfusion. The representative photographs of mid-ventricular cross-sections and the TUNEL-positive cells in the myocardial slices from mice are shown in Figure 1. The white zone (unstained by Evans Blue and TTC) was determined as the infarct area. The extent of infarct myocardium was presented as a percentage of INF/LV. We found that myocardial I/R injury resulted in a sharp increase in INF/LV percentage compared with Sham group. Furthermore, as shown in Figure 1B, apoptotic index was significantly higher in the myocardial I/R group than the Sham group. These results suggested that the *in vivo* model of myocardial I/R injury was successful.

**Figure 2:**
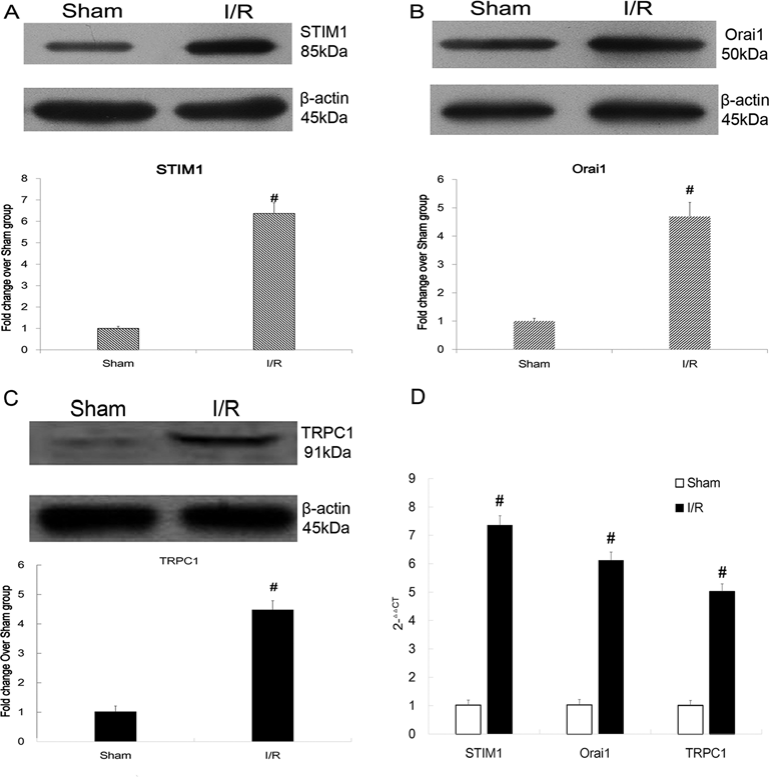
Up-regulated expression of STIM1/Orai1/TRPC1 in mice subjected to Sham operation and myocardial I/R injury. (**A–C**) Levels of STIM1, Orai1, and TRPC1 protein analyzed by Western blot. The protein levels of STIM1, Orai1, and TRPC1 increased 5.95-fold (*t* = − 148.388, *P*<0.001), 4.65-fold (*t* = −167.414, *P*<0.001), and 4.19-fold (*t* = −204.411, *P*<0.001) respectively, compared with the Sham group; **#***P*<0.05 vs Sham operation. (**D**) Levels of STIM1, Orai1, and TRPC1 mRNA analyzed by RT-PCR. The mRNA levels of STIM1, Orai1, and TRPC1 increased 7.03-fold (*t* = −142.880, *P*<0.001), 5.94-fold (*t* = −87.303, *P*<0.001), and 5.05-fold (*t* = −66.339, *P*<0.001) respectively, compared with the Sham group (*n* = 10 in each group); **#***P*<0.05 vs Sham operation. The results shown are mean ± S.E.M. from three independent experiments.

We then detected the expression of STIM1, Orai1, and TRPC1 by both RT-PCR and Western blot. As shown in Figure 2A–C, after myocardial I/R, the protein levels of STIM1, Orai1, and TRPC1 significantly increased compared with the Sham group. As illustrated in Figure 2D, the mRNA levels of STIM1, Orai1, and TRPC1 also dramatically increased compared with the Sham group.

### Expression of STIM1, Orai1, and TRPC1 in H9C2 cardiomyocytes after H/R injury

**Figure 3:**
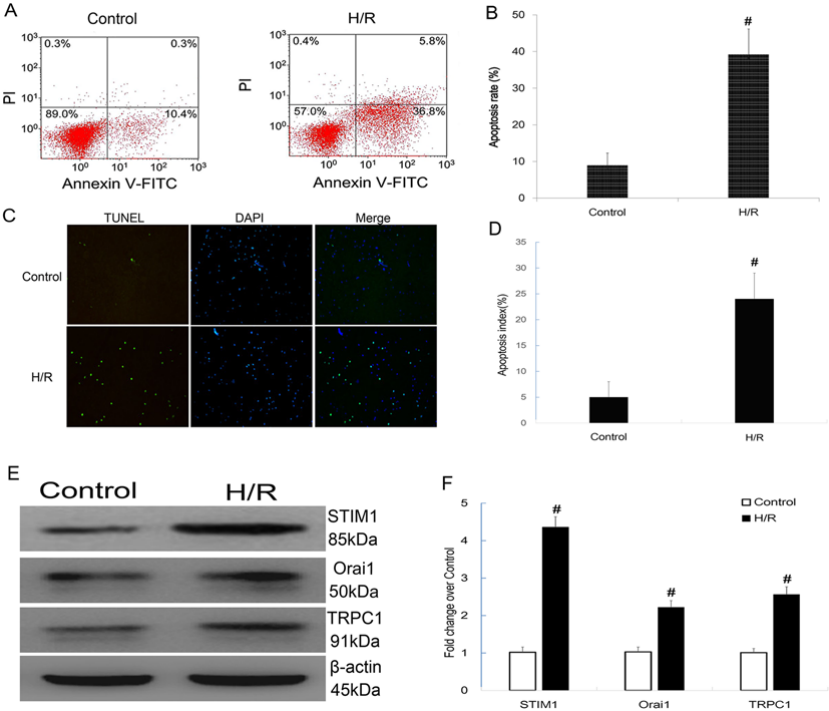
Determination of apoptosis and expression of STIM1/Orai1/TRPC1 in H9C2 cardiomyocytes subjected to Control and H/R injury. (**A** and **B**) Representative photographs of flow cytometry using Annexin V/PI double staining in cardiomyocytes underwent Control and H/R. Apoptosis rate significantly increased following H/R in cardiomyocytes(34.86 ± 6.16% vs 9.16 ± 4.07%, *P*<0.001, *n* = 6 in each group); **#***P*<0.05 vs Control; (**C** and **D**) Representative photographs of TUNEL assay in cardiomyocytes underwent Control and H/R. Apoptosis index significantly increased following H/R in cardiomyocytes (26.56 ± 3.35% vs 5.57 ± 1.63%, *P*<0.001, *n* = 6 in each group); **#***P*<0.05 vs Control. (**E** and **F**) Levels of STIM1, Orai1, and TRPC1 protein analyzed by Western blot. The protein levels of STIM1, Orai1, and TRPC1 increased 4.25-fold (*t* = −27.717, *P*<0.001), 2.26-fold (*t* = −15.418, *P*<0.001), and 2.51-fold (*t* = −19.925, *P*<0.001) respectively, compared with the Control group; **#***P*<0.05 vs Control. The results shown are mean± S.E.M. from three independent experiments.

To establish the *in vitro* hypoxia/reoxygenation (H/R) injury model, H9C2 cardiomyocytes underwent hypoxia for 3 h then reoxygenation for 16 h. The validity of H/R model was evaluated by flow cytometry using Annexin V/PI double staining and TUNEL assay. The results showed that the apoptotic rate measure by flow cytometry in the H/R injury group was significantly higher than the control group (Figure 3A,B). Furthermore, the apoptotic index assessed by TUNEL in the H/R injury group was significantly higher than the control group (Figure 3C,D).

Then protein levels of STIM1, Orai1, and TRPC1 were detected by Western blot. As shown in Figure 3E,F, after H/R injury, the protein levels of STIM1, Orai1, and TRPC1 remarkably increased compared with the Control group.

### Transfection of specific SiRNA inhibited STIM1 expression

**Figure 4:**
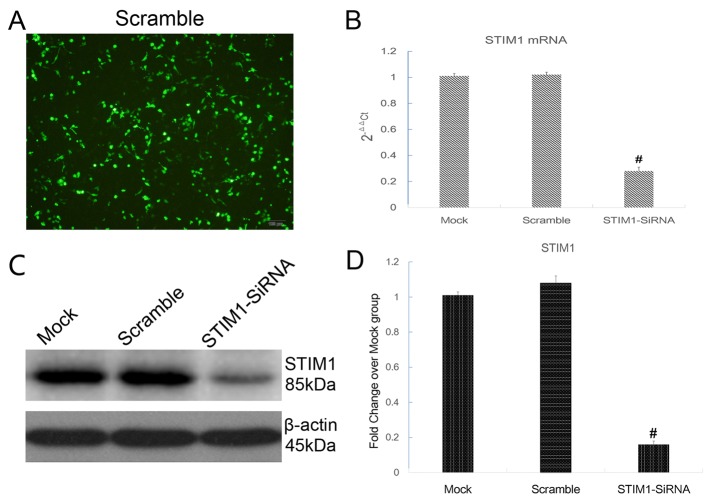
Transfection of specific SiRNA inhibited STIM1 expression in H9C2 cardiomyocytes. (**A**) Transfection efficiency (assessed by FAM-labeled Scramble SiRNA) was 67.6 ± 8.4%. (**B**) Levels of STIM1 mRNA was determined by RT-PCR. Results were expressed as fold change over control group; **#***P*<0.05 vs Control. (**C** and **D**) Levels of STIM1 protein was determined by Western blot. Results were expressed as fold change over control group; **#***P*<0.05 vs Control. The results shown are mean ± S.E.M. from three independent experiments.

**Figure 5:**
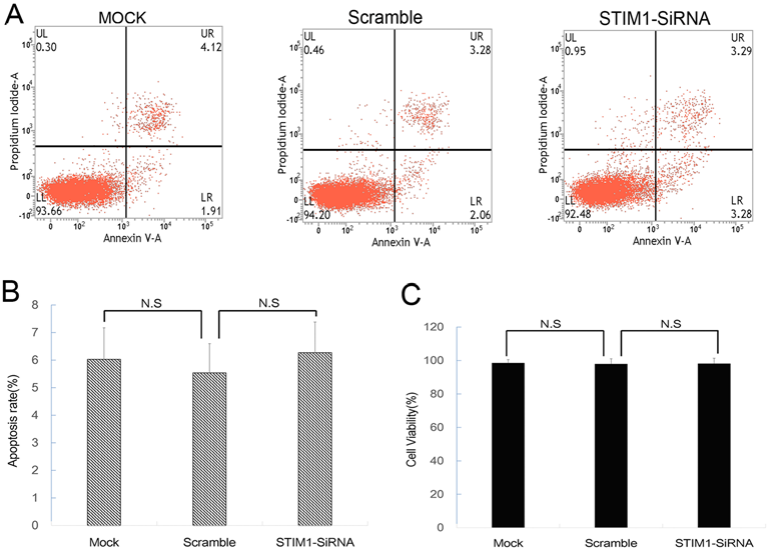
Effect of SiRNA transfection on apoptosis and cell viability of H9C2 cardiomyocytes. (**A** and **B**) Representative photographs of flow cytometry using Annexin V/PI double staining in cardiomyocytes 48 h after transfection. (**C**) Cell viability assessed by MTT assay in cardiomyocytes 48 h after transfection. The results shown are mean± S.E.M. from three independent experiments.

To determine the interaction between up-regulation of STIM1 and H/R injury, STIM1 was suppressed by transfecting with specific SiRNA. Scramble SiRNA labeled with FAM (Thermo Fisher scientific, Shanghai, China) was used to establish the negative control and evaluate the transfection efficacy. Cells transfected with Lipofectamine reagent only were considered as a negative control (Mock group). The transfection efficacy was 67.6 ± 8.4% as illustrated in Figure 4A. To further determine the inhibition efficacy, expression of STIM1 was detected 48h after transfection. As shown in Figure 4B,D, specific SiRNA against STIM1 significantly decreased mRNA and protein expression of STIM1. Furthermore, there were no significant differences in the apoptosis rate and cell viability among the groups before being subjected to H/R injury (Figure 5), suggesting no toxicity of SiRNA and transfection reagent on H9C2 cardiomyocytes.

### Suppression of STIM1 decreased apoptosis in H9C2 cardiomyocytes induced by H/R

**Figure 6:**
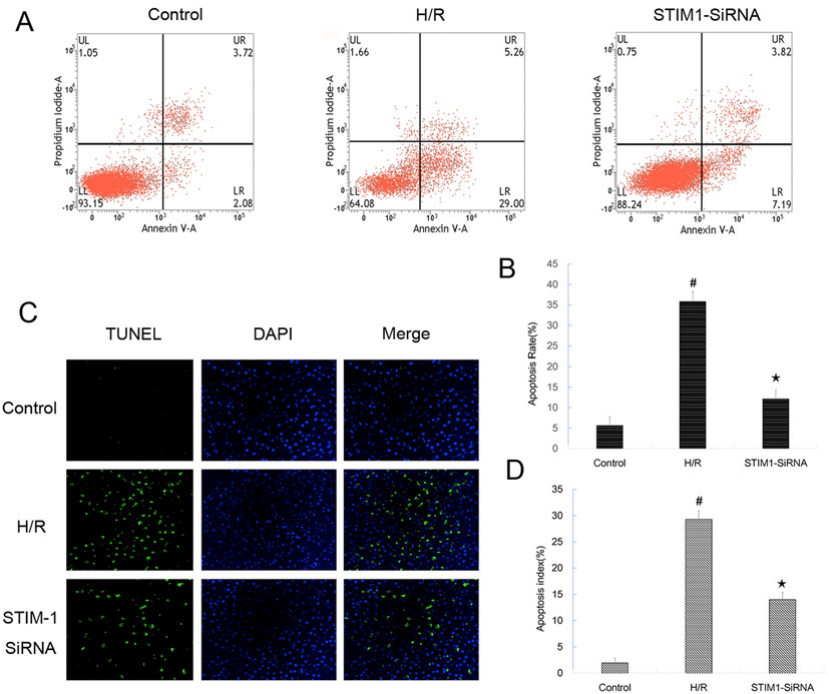
Suppression of STIM1 decreased apoptosis in H9C2 cardiomyocytes induced by H/R injury. (**A** and **B**) Apoptosis rate assessed by flow cytometry using Annexin V/PI double staining in H9C2 cardiomyocytes subjected to Control, H/R, and SiRNA + H/R. The apoptotic rate in the H/R injury group was significantly higher than the control group (32.91 ± 4.45% vs 6.28 ± 3.19% respectively, *F* = 83.388, *P*<0.001, *n* = 6 in each group). In addition, knock-down of STIM1 before H/R decreased the apoptosis rate compared with that of the cells that underwent H/R alone (12.55 ± 3.44% vs 32.91 ± 4.45%, *F* = 83.388, *P*<0.001, *n* = 6 in each group). (**C** and **D**) Apoptosis index assessed by TUNEL assay in H9C2 cardiomyocytes subjected to Control, H/R, and SiRNA + H/R. The apoptosis index in the H/R injury group was significantly higher than the control group (25.94 ± 5.99% vs 2.84 ± 1.29%, *F* = 52.788, *P*<0.001). Furthermore, the apoptosis index in STIM1 knock-down group was significantly lower than H/R group (11.55 ± 2.98% vs 25.94 ± 5.99%%, *F* = 83.388, *P*<0.001); **#***P*<0.05 vs control; ★*P*<0.05 vs H/R group. The results shown are mean ± S.E.M. from three independent experiments.

Then the impact of STIM1 knock-down on apoptosis of H9C2 cardiomyocytes after H/R was evaluated by both flow cytometry and TUNEL assay. The result showed that the apoptotic rate in the H/R injury group was significantly higher than the control group (Figure 6A,B). In addition, the apoptosis index assessed by TUNEL in the H/R injury group was also significantly higher than the control group (Figure 6C,D). Knock-down of STIM1 before H/R decreased the apoptosis rate compared with that of the cells that underwent H/R alone (Figure 6A,B). Furthermore, the apoptosis index of H9C2 cells in STIM1 knock-down group was also significantly less than that of the cells that underwent H/R alone (Figure 6C,D).

### Suppression of STIM1 by RNAi reduced intracellular Ca^2+^ accumulation induced by H/R in H9C2 cardiomyocytes

**Figure 7:**
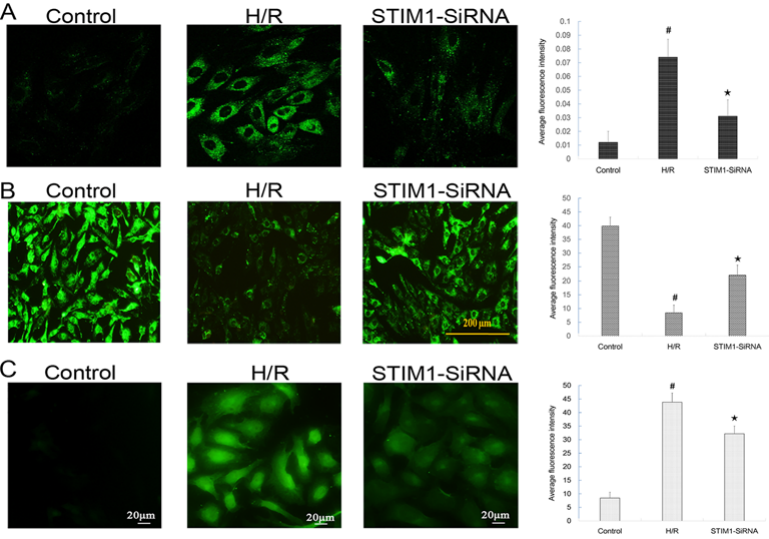
Suppression of STIM1 by RNAi reduced intracellular Ca^2+^ accumulation, mPTP opening and ROS generation induced by H/R in H9C2 cardiomyocytes. (**A**) Intracellular Ca^2+^ levels was detected by laser scanning confocal microscopy 48 h following transfection. Intensity of Ca^2+^ fluorescence marked by fluo-3/AM under confocal microscope significantly enhanced (4.7-fold than the Control, *F* = 19.902, *P*<0.001) after H/R and remarkably decreased, but still stronger than control group cells; **#***P*<0.05 vs control; ★*P*<0.05 vs H/R group. (**B**) The opening of the transient mPTP was assessed by coloading with calcein AM and CoCl_2_ in H9C2 cardiomyocytes. Opening of mPTP significantly enhanced after H/R and remarkably decreased, but still stronger than control group cells; **#***P*<0.05 vs control; ★*P*<0.05 vs H/R group. (**C**) ROS generation was evaluated using the dye DCFH-DA in H9C2 cardiomyocytes. ROS generation significantly increased after H/R and remarkably decreased, but still stronger than control group cells; **#***P*<0.05 vs control; ★*P*<0.05 vs H/R group. The results shown are mean ± S.E.M. from three independent experiments.

Ca^2+^ overload is known to be an important mechanism responsible for MIRI. To examine whether intracellular Ca^2+^ accumulation was affected by blocking STIM1, the intensity of Ca^2+^ fluorescence marked by fluo-3/AM was measured under confocal microscope after the H/R procedure. We found that the intensity was increased significantly after H/R (Figure 7A) compared with control group. The result indicated that intracellular Ca^2+^ accumulation was increased in H/R treated H9C2 cells. Besides, the intensity of Ca^2+^ fluorescence was significantly decreased in transfected cells after the H/R, when compared with that in those without transfection (Figure 7A).

### Suppression of STIM1 by RNAi inhibited mPTP opening and ROS generation induced by H/R in H9C2 cardiomyocytes

Due to mitochondria plays a central role in apoptosis and Ca^2+^ overload is believed to prime the mPTP to open at the stage of myocardial reperfusion, the mPTP opening was evaluated in H9C2 cardiomyocytes after H/R. The occurrence of mPTP opening in intact cells was investigated by monitoring the fluorescence of mitochondrial-entrapped calcein with the calcein-AM and CoCl_2_ coloading method. As shown in Figure 7B, compared with the control group, the fluorescence intensity of mitochondrial calcein was significantly decreased in H/R group, indicating that the extent of mPTP opening was enhanced following H/R. Moreover, knock-down of STIM1 markedly increased the fluorescence intensity compared with the H/R, implying that knock-down of STIM1 inhibited H/R-induced mPTP opening in H9c2 cardiomyocytes.

As oxidative stress plays an important role in the myocardial I/R injury, the effect of STIM1 knock-down upon ROS generation was evaluated using the dye DCFH-DA. As shown in Figure 7C, the fluorescence intensity of DCF significantly raised in H/R group, indicating that the ROS level increased following H/R. Furthermore, knock-down of STIM1 markedly reduced the fluorescence intensity compared with the H/R, suggesting that knock-down of STIM1 decreased ROS generation.

### Suppression of STIM1 by RNAi increased the Bcl-2/Bax ratio, decreased Orai1/TRPC1, and cleaved caspase-3 expression

**Figure 8:**
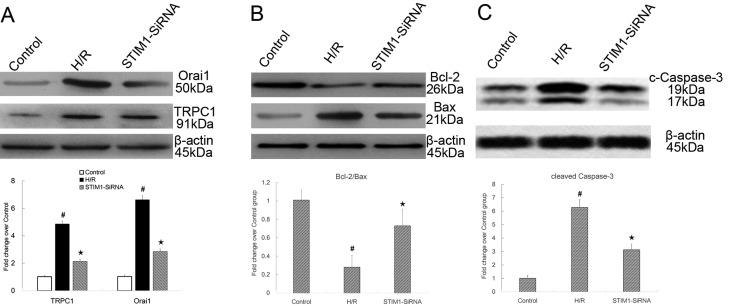
Suppression of STIM1 by RNAi increased the Bcl-2/Bax ratio, decreased TRPC1, and cleaved caspase-3 expression. (**A**) The levels of Orai1 and TRPC1 protein increased in H9C2 cardiomyocytes after H/R and significantly decreased in H9C2 cardiomyocytes transfected with STIM1–SiRNA after H/R. The expression of Orai1 significantly increased in H9C2 cells that underwent H/R (7.2-fold vs the control group, χ^2^ = 15.189, *P*=0.001), and this increase was blocked by suppression of STIM1 (0.39-fold vs the H/R group, *P*=0.001). In addition, the expression of TRPC1 significantly increased in H9C2 cells that underwent H/R (4.4-fold vs the control group, χ^2^ = 15.158, *P*=0.001), and this increase was also blocked by suppression of STIM1 (0.44-fold vs the H/R group, *P*=0.001); **#***P*<0.05 vs control; ★ *P*<0.05 vs H/R group. (**B**) The Bcl-2/Bax ratio was significantly decreased in the H/R group. Suppression of STIM1 by RNAi resulted in a noticeable increase in the Bcl-2/Bax ratio compared with the H/R group. The Bcl-2/Bax ratio was significantly decreased in the H/R group (0.25-fold in H/R group vs the control group, *F* = 143.399, *P*<0.001). However, suppression of STIM1 by RNAi resulted in a noticeable increase in the Bcl-2/Bax ratio compared with that of the H/R group (2.58-fold vs the H/R group, *P*<0.001); **#***P*<0.05 vs control; ★*P*<0.05 vs H/R group. (**C**) The levels of cleaved caspase-3 protein significantly increased in H9C2 cells underwent H/R and this increase was blocked by suppression of STIM1. The expression of cleaved caspase-3 significantly increased in H9C2 cells that underwent H/R (6.12-fold vs the control group, *F* = 214.178, *P*<0.001), and this increase was blocked by suppression of STIM1 (0.45-fold vs the H/R group, *P*<0.001); **#***P*<0.05 vs control; ★*P*<0.05 vs H/R group. The results shown are mean ± S.E.M. from three independent experiments.

To investigate the effects of STIM1 suppression on the expression the downstream molecules, Orai1 and TRPC1 protein levels were measured with Western blot. As shown in Figure 8A, the expression of Orai1 significantly increased in H9C2 cells that underwent H/R, and this increase was blocked by suppression of STIM1. In addition, the expression of TRPC1 significantly increased in H9C2 cells that underwent H/R, and this increase was also blocked by suppression of STIM1.

Bcl-2 and Bax are known to be involved in the control of apoptotic pathways. Particularly, the ratio of Bcl-2 to Bax protein determines whether cells will die via apoptosis or be protected from it. As shown in Figure 8B, the Bcl-2/Bax ratio was significantly decreased in the H/R group. However, suppression of STIM1 by RNAi resulted in a noticeable increase in the Bcl-2/Bax ratio compared with that of the H/R group. As illustrated in Figure 8C, the expression of cleaved caspase-3 significantly increased in H9C2 cells that underwent H/R, and this increase was blocked by suppression of STIM1.

## Discussion

The present study found that myocardial I/R injury resulted in the up-regulation of STIM1/Orai1/TRPC1 in the *in vivo* mouse model. The *in vitro* study then revealed that knock-down of STIM1 with specific SiRNA attenuated H/R-induced apoptosis in H9C2 cardiomyocytes by reducing intracellular Ca^2+^ accumulation and inhibiting mPTP, thereby altering the expression of TRPC1 as well as apoptosis-related proteins including Bcl-2, Bax, and caspase-3. These results suggested the therapeutic potential of STIM-1/Orai1 for myocardial I/R injury.

In recent years, STIM1 has been shown to act as an ER sensor and mediate SOCE, a major mechanism of Ca^2+^ influx in nearly all excitable cells, including cardiomyocytes. Previous studies provided substantial evidence that STIM1-mediated SOCE plays a key role in mediating cardiomyocyte hypertrophy, both *in vitro* and *in vivo*. In addition, pharmacological inhibition of SOCE has been confirmed to attenuate Ca^2+^ overload in cardiac microvascular endothelial cells and cardiomyocytes [20,21], suggesting the potential contribution of SOCE to Ca^2+^ overload in the process of ischemia/reperfusion injury. These previous evidence helped the current study to put forward ideas in cardiomyocytes suffering from I/R injury. The first problem needed to be resolved was expression of STIM1/Orai1 at reperfusion stage. In present study, we found that in a mouse model of myocardial I/R injury, along with extended infarct size and increased apoptosis, the levels of STIM1, Orai1 and TRPC1 mRNA, and protein increased dramatically compared with Sham group. Similarly, the protein levels of STIM1, Orai1, and TRPC1 significantly increased in cultured H9C2 cardiomyocytes in a simulated H/R injury model. These results provided a hint that up-regulation of STIM1/Orai1/TRPC1 might participate in the pathogenesis of myocardial I/R injury.

It is well known that apoptosis is one of the major pathogenic mechanisms underlying I/R injury and previous studies have demonstrated that blockade of apoptosis could effectively decrease the loss of contractile cells, minimize I/R-induced myocardial injury and, therefore, slowdown or even prevent the occurrence of heart failure. Although STIM1/Orai1/TRPC1 was proven to be up-regulated during myocardial reperfusion, it is unknown whether down-regulation of STIM1/Orai1 prevents the heart against I/R injury. Up-regulation of STIM1/Orai1 has been reported to trigger apoptosis in several cells. For example, down-regulation of STIM1/Orai1 counteracts the apoptosis of nasopharyngeal carcinoma cells induced by NaBu [22]. Similarly, down-regulated SOCE by SiRNA targeting STIM1 significantly inhibited soft substrate-induced epithelial cell apoptosis [23]. To investigate the effect of down-regulation of STIM1 in the context of myocardial I/R injury, we performed *in vitro* studies in which H9C2 cardiomyocytes was subjected to H/R injury to mimic myocardial I/R injury and STIM1 was knocked down with specific SiRNA. The results revealed that knock-down of STIM1 significantly inhibited apoptosis in H9C2 cardiomyocytes subjected to H/R as evidenced by both TUNEL assay and flow cytometry using Annexin V/PI staining. The present study also demonstrated that suppression of STIM1 up-regulated Bcl-2 and down-regulated Bax and cleaved caspase-3 protein expression levels in cultured H9C2 cardiomyocytes. Therefore, as above, it was suggested that inhibiting apoptosis of cardiomyocytes was one of the important mechanisms by which suppression of STIM1 exerted cardioprotective effects during H/R injury.

Abundant evidence from previous studies have demonstrated that a rise in intracellular Ca^2+^([Ca^2+^]_i_) preceded irreversible myocardial injury and drugs or protocols that block the rise in [Ca^2+^]_i_ could reduce or delayed myocardial death. The molecular mechanisms underlying [Ca^2+^]_i_ accumulation has not been fully elucidated. Both the Na^+^–H^+^ exchanger (NHE) and the voltage gated L-type calcium channel have been demonstrated to be the primary mechanisms leading to lethal Ca^2+^ overload during the reperfusion stage. Previous study revealed that activation of STIM1 in cardiomyocytes led to enhanced Ca^2+^ entry [9]. This increase in intracellular Ca^2+^ mediated by SOCE has been demonstrated critical elements in the upstream, Ca^2+^-dependent control of pathological cardiac hypertrophy [9,24]. In addition, in cardiac microvascular endothelial cells exposed to reperfusion conditions, the enhanced Ca^2+^ overload is due to SOC-mediated Ca^2+^ influx [20,21]. In agreement with previous studies, we observed a raise in Ca^2+^ concentration in cultured H9C2 cardiomyocytes after being exposed to H/R. Moreover, the increase in [Ca^2+^]_i_ accumulation in H9C2 cardiomyocytes induced by H/R injury was significantly attenuated after suppression of STIM1. These results suggest that Ca^2+^ accumulation mediated by STIM1 may contribute to Ca^2+^ overload following H/R injury.

Increase in ROS is also a key mediator of myocardial I/R injury. Indeed, STIM1-mediated Ca^2+^ influx has been shown to increase mitochondrial ROS production in many different cells [25–27]. Similarly, the present study observed significant ROS increase in H9C2 cells during H/R injury. However, H/R injury induced ROS generation was attenuated when STIM1 was knocked down by SiRNA, suggesting that ROS generation may contribute to the H/R injury in H9C2 cardiomyocytes. Excessive ROS generation triggers the opening of mPTP, which initiates death pathways. Correspondingly, our study also showed prompted mPTP opening in H9C2 cardiomyocytes after H/R. Furthermore, suppression of STIM1 by SiRNA obviously reversed H/R injury induced mPTP opening during H/R. Taken together, these data indicate that the inhibition of mPTP opening triggered by excessive ROS generation may independently contribute to the cardioprotective effects of STIM1 suppression in cardiomyocytes suffering from H/R injury.

In summary, our results show that both STIM1 and its target molecules Orai1/TRPC1 were up-regulated in the context of myocardial I/R injury. Furthermore, STIM1-induced intracellular Ca^2+^ accumulation may involve in ROS production and then induce the opening of mPTP, finally lead to cell death and apoptosis in H9C2 cardiomyocytes subjected to H/R injury. Our findings provide a novel cellular mechanism involved in the pathophysiology of myocardial I/R injury.

The present study has some limitations. First, in the present study, we used the H9C2 cardiomyocytes to establish the *in vitro* H/R model. However, the extent to which H9C2 cells can accurately mimic the H/R responses of primary cardiac myocytes has not yet been fully established. In addition, the present study on H9c2 cells was designed to uncover the potential signaling mechanisms impacting on Ca^2+^ accumulation after I/R. Due to the complexity of the *in vivo* environment, especially after I/R, results from H/R study are not sufficient to explain the exact function of STIM1/Orai1/TRPC1 in Ca^2+^ overload post I/R. But this study suggested at least a feasibility of carrying such study on mammalian myocardial cells, and provided a theoretical and experimental basis to put these findings into further understanding of the mechanisms underlying the accumulation of calcium via STIM1/Orai1 signaling pathway in the myocardial systems under both physiological and pathological conditions.

## Abbreviations

AAR: area-at-risk
ER: endoplasmic reticulum
H/R: hypoxia/reoxygenation
INF: infarct size
I/R: ischemia/reperfusion
LAD: left ascending artery
MIRI: myocardial ischemia/reperfusion injury
mPTP: mitochondrial permeability transition pore
SOCE: store-operated Ca^2+^ entry
STIM: stromal interaction molecule 1

## References

[1] Yu J., Zhang X. and Zhang Y. (2017) Astragaloside attenuates myocardial injury in a rat model of acute myocardial infarction by upregulating hypoxia inducible factor-1 alpha and Notch1/Jagged1 signaling. Mol. Med. Rep. 15, 4015–4020 28487976 10.3892/mmr.2017.6522 PMC5436283

[2] Yellon D.M. and Hausenloy D.J. (2007) Mechanisms of disease: myocardial reperfusion injury. N. Engl. J. Med. 357, 1121–1135 17855673 10.1056/NEJMra071667

[3] Brooks W.W., Conrad C.H. and Morgan J.P. (1995) Reperfusion induced arrhythmias following ischaemia in intact rat heart: role of intracellular calcium. Cardiovasc. Res. 29, 536–542 7796448

[4] Murphy E. and Steenbergen C. (2008) Mechanisms underlying acute protection from cardiac ischemia-reperfusion injury. Physiol. Rev. 88, 581–609 18391174 10.1152/physrev.00024.2007 PMC3199571

[5] Garcia-Dorado D., Ruiz-Meana M., Inserte J., Rodriguez-Sinovas A. and Piper H.M. (2012) Calcium-mediated cell death during myocardial reperfusion. Cardiovasc. Res. 94, 168–180 22499772 10.1093/cvr/cvs116

[6] Pang Y., Hunton D.L., Bounelis P. and Marchase R.B. (2002) Hyperglycemia inhibits capacitative calcium entry and hypertrophy in neonatal cardiomyocytes. Diabetes 51, 3461–3467 12453900 10.2337/diabetes.51.12.3461

[7] Collins H.E., Zhu-Mauldin X., Marchase R.B. and Chatham J.C. (2013) STIM1/Orai1-mediated SOCE: current perspectives and potential roles in cardiac function and pathology. Am. J. Physiol. Heart Circ. Physiol. 305, H446–H458 23792674 10.1152/ajpheart.00104.2013 PMC3891250

[8] Hogan P.G., Lewis R.S. and Rao A. (2010) Molecular basis of calcium signaling in lymphocytes: STIM and ORAI. In Annual Review of Immunology (Paul W.E., Littman D.R. and Yokoyama W.M., eds), vol. 28, pp. 491–533 10.1146/annurev.immunol.021908.132550 PMC2861828 20307213

[9] Correll R.N., Goonasekera S.A., van Berlo J.H., Burr A.R., Accornero F., Zhang H. (2015) STIM1 elevation in the heart results in aberrant Ca2+ handling and cardiomyopathy. J. Mol. Cell Cardiol. 87, 38–47 26241845 10.1016/j.yjmcc.2015.07.032 PMC4637225

[10] Hulot J.S., Fauconnier J., Ramanujam D., Chaanine A., Aubart F., Sassi Y. (2011) Critical role for stromal interaction molecule 1 in cardiac hypertrophy. Circulation 124, 796–U109 21810664 10.1161/CIRCULATIONAHA.111.031229 PMC3428713

[11] Luo X., Hojayev B., Jiang N., Wang Z.V., Tandan S., Rakalin A. (2012) STIM1-dependent store-operated Ca2+ entry is required for pathological cardiac hypertrophy. J. Mol. Cell Cardiol. 52, 136–147 22108056 10.1016/j.yjmcc.2011.11.003 PMC3247164

[12] Liu J., Pang Y., Chang T., Bounelis P., Chatham J.C. and Marchase R.B. (2006) Increased hexosamine biosynthesis and protein O-GlcNAc levels associated with myocardial protection against calcium paradox and ischemia. J. Mol. Cell Cardiol. 40, 303–312 16337959 10.1016/j.yjmcc.2005.11.003

[13] Chatham J.C. and Marchase R.B. (2010) The role of protein O-linked β-N-acetylglucosamine in mediating cardiac stress responses. Biochim. Biophys. Acta 1800, 57–66 19607882 10.1016/j.bbagen.2009.07.004 PMC2814923

[14] Oyama J., Blais C., Liu X.L., Pu M.Y., Kobzik L., Kelly R.A. (2004) Reduced myocardial ischemia-reperfusion injury in toll-like receptor 4-deficient mice. Circulation 109, 784–789 14970116 10.1161/01.CIR.0000112575.66565.84

[15] Wang X., He F., Liao Y., Song X., Zhang M., Qu L. (2013) Baicalin pretreatment protects against myocardial ischemia/reperfusion injury by inhibiting mitochondrial damage-mediated apoptosis. Int. J. Cardiol. 168, 4343–4345 23725813 10.1016/j.ijcard.2013.05.077

[16] Ren B.H., Shen Y., Shao H.T., Qian J.J., Wu H.W. and Jing H. (2007) Brain natriuretic peptide limits myocardial infarct size dependent of nitric oxide synthase in rats. Clin. Chim. Acta 377, 83–87 17026975 10.1016/j.cca.2006.08.027

[17] He F., Xu B.L., Chen C., Jia H.J., Wu J.X., Wang X.C. (2016) Methylophiopogonanone A suppresses ischemia/reperfusion-induced myocardial apoptosis in mice via activating PI3K/Akt/eNOS signaling pathway. Acta Pharmacol. Sin. 37, 763–771 27063216 10.1038/aps.2016.14 PMC4954762

[18] Zhou S.S., He F., Chen A.H., Hao P.Y. and Song X.D. (2012) Suppression of rat Frizzled-2 attenuates hypoxia/reoxygenation-induced Ca2+ accumulation in rat H9c2 cells. Exp. Cell Res. 318, 1480–1491 22510436 10.1016/j.yexcr.2012.03.030

[19] Lee S., Kim K., Kim Y.H., Chung M.H., Kang I., Ha J. (2011) Preventive role of Propofol in hypoxia/reoxygenation-induced apoptotic H9c2 rat cardiac myoblast cell death. Mol. Med. Rep. 4, 351–356 21468576 10.3892/mmr.2011.432

[20] Kojima A., Kitagawa H., Omatsu-Kanbe M., Matsuura H. and Nosaka S. (2010) Ca2+ paradox injury mediated through TRPC channels in mouse ventricular myocytes. Br. J. Pharmacol. 161, 1734–1750 20718730 10.1111/j.1476-5381.2010.00986.x PMC3010579

[21] Peters S.C. and Piper H.M. (2007) Reoxygenation-induced Ca2+ rise is mediated via Ca2+ influx and Ca2+ release from the endoplasmic reticulum in cardiac endothelial cells. Cardiovasc. Res. 73, 164–171 17097624 10.1016/j.cardiores.2006.09.015

[22] Huang W., Ren C.P., Huang G.L., Liu J., Liu W.D., Wang L. (2017) Inhibition of store-operated Ca2+ entry counteracts the apoptosis of nasopharyngeal carcinoma cells induced by sodium butyrate. Oncol. Lett. 13, 921–929 28356979 10.3892/ol.2016.5469 PMC5351044

[23] Chiu W.T., Tang M.J., Jao H.C. and Shen M.R. (2008) Soft substrate up-regulates the interaction of STIM1 with store-operated Ca2+ channels that lead to normal epithelial cell apoptosis. Mol. Biol. Cell 19, 2220–2230 18337467 10.1091/mbc.E07-11-1170 PMC2366837

[24] Voelkers M., Salz M., Herzog N., Frank D., Dolatabadi N., Frey N. (2010) Orai1 and Stim1 regulate normal and hypertrophic growth in cardiomyocytes. J. Mol. Cell Cardiol. 48, 1329–1334 20138887 10.1016/j.yjmcc.2010.01.020 PMC5511312

[25] Mungai P.T., Waypa G.B., Jairaman A., Prakriya M., Dokic D., Ball M.K. (2011) Hypoxia triggers AMPK activation through reactive oxygen species-mediated activation of calcium release-activated calcium channels. Mol. Cell. Biol. 31, 3531–3545 21670147 10.1128/MCB.05124-11 PMC3165558

[26] Kim M.S., Yang Y.M., Son A., Tian Y.S., Lee S.I., Kang S.W. (2010) RANKL-mediated reactive oxygen species pathway that induces long lasting Ca2+ Oscillations essential for osteoclastogenesis. J. Biol. Chem. 285, 6913–6921 20048168 10.1074/jbc.M109.051557 PMC2844141

[27] Gandhirajan R.K., Meng S., Chandramoorthy H.C., Mallilankaraman K., Mancarella S., Gao H. (2013) Blockade of NOX2 and STIM1 signaling limits lipopolysaccharide-induced vascular inflammation. J. Clin. Invest. 123, 887–902 23348743 10.1172/JCI65647 PMC3561818

